# Bariatric surgery in morbidly obese individuals affects plasma levels of protein C and thrombomodulin

**DOI:** 10.1007/s11239-018-1744-9

**Published:** 2018-09-26

**Authors:** Gersina Rega-Kaun, Christoph Kaun, Benjamin Ebenbauer, Gerlinde Jaegersberger, Manfred Prager, Johann Wojta, Philipp J. Hohensinner

**Affiliations:** 1 Department of Internal Medicine II, Medical University of Vienna, Vienna, Austria; 2 5th Medical Department for Endocrinology and Rheumatology, Wilhelminen Hospital, Vienna, Austria; 3 Ludwig Boltzmann Cluster for Cardiovascular Research, Vienna, Austria; 4 Department of Surgery, Hospital Hietzing, Vienna, Austria; 5 Department of Surgery, Hospital Oberwart, Vienna, Austria; 6 Core Facilities, Medical University of Vienna, Vienna, Austria

**Keywords:** Bariatric surgery, Protein C, Thrombomodulin

## Abstract

Obesity is associated with a prothrombotic milieu and an increased risk for thrombotic events. Bariatric surgery is the most effective treatment for obesity resulting in dramatic weight loss and reduced inflammation and extrinsic coagulation pathway activation. Blood samples were drawn from 60 patients undergoing Roux-en-Y gastric bypass surgery before and 1 year after the intervention. Protein C (PC), activated PC (APC), soluble thrombomodulin (TM), soluble E-selectin (E-Sel), prothrombin time (PT) and activated partial thromboplastin time (aPTT) were evaluated. Both PC (187.4 ± 64.5% before surgery to 118.1 ± 48% 1 year after surgery, p < 0.001) and APC (138.7 ± 64.4% before surgery to 69.1 ± 65.7% after surgery, p < 0.001) were reduced following surgical intervention. TM showed a similar behavior with a reduction of soluble TM after the procedure from 5.7 ± 2.6 to 3.2 ± 1.4 ng/ml (p < 0.001). Similarly, soluble E-Sel was reduced after surgery from 26.6 ± 12.7 to 5.5 ± 4.1 ng/ml (p < 0.001). In contrast, aPTT was not shortened but slightly increased from 29.1 ± 4.8 s. before surgery to 31 ± 4.4 s. (p = 0.001) after surgery and levels of PT were reduced after surgery to 89.6 ± 15.5% from an initial 97.5 ± 13.5% (p < 0.001). In conclusion, we demonstrate a reduction of PC and APC 1 year after bariatric surgery accompanied by a reduction in soluble TM and soluble E-Sel. The reduction of PC and APC is not paralleled by a reduction but in contrast by a prolongation of aPTT suggesting a compensatory upregulation of PC during obesity. The reduction of TM and E-Sel might hint towards an improved endothelial function in this cohort of patients.

## Highlights

- Bariatric surgery changes the coagulatory profile of morbidly obese patients
- Plasma levels of protein C, activated protein C and soluble thrombomodulin were significantly reduced 1 year after surgery
- Prothrombin time was significantly shortened and activated partial thromboplastin time was significantly prolonged 1 year after surgery

## Introduction

Obesity as a major health problem worldwide is rising with a yearly mean body mass index (BMI) increase of 0.4–0.5 kg/m^2^ [1]. Obesity causes an increase in proinflammatory cytokines and premature aging [2, 3]. In addition, obese patients show changes in their procoagulant state including increased risk of venous and arterial thromboembolism and hypercoagulability after injury [4–7]. Overall, obese patients have higher plasma concentrations of pro-thrombotic factors as compared to non-obese patients [8]. Guideline recommendations for morbidly obese patients include bariatric surgery after failure of weight loss in a structured conservative program for patients starting at a BMI > 40 or 35 kg/m^2^ with secondary disease [9]. Bariatric surgery leads to a massive weight loss including obesity related changes in inflammation and coagulation.

The coagulation cascade can be differentiated into an extrinsic and an intrinsic pathway. The extrinsic pathway is driven by tissue factor (TF) which can be inhibited by its natural inhibitor TF pathway inhibitor [10]. TF is usually located on circulating microvesicles and initiates coagulation via activation of factor VII. Previous reports already suggested a reduction of circulating TF after weight loss and a decrease in microvesicle-associated TF in morbidly obese patients after bariatric surgery [11, 12].

The intrinsic pathway is initiated by factor XII. Activated protein C (APC) is the active anticoagulant enzyme formed by activation of protein C (PC) for the intrinsic coagulation pathway [13]. The activation of PC is dependent on thrombomodulin (TM) [14]. Both cell associated and soluble form of TM promote specific effects in coagulation through generation of APC. Increased plasma levels of TM have been described in patients with disseminated intravascular coagulation syndrome, pulmonary thromboembolism and hepatic failure [15–17]. The loss of TM from endothelial cell surfaces is thought to contribute to increased risk of thrombosis.

To better understand components of the intrinsic coagulation cascade in obesity and to determine the effect of massive weight loss on those parameters we analyzed levels of PC, APC and TM in patients undergoing Roux-en-Y gastric bypass surgery. In addition, we determined the overall coagulatory state of the patient using activated partial thromboplastin time (aPTT) and prothrombin time (PT).

## Methods

### Patient recruitment and sampling

In total, 60 patients were included in the study who were selected to undergo recommended Roux-en-Y gastric bypass surgery. Our cohort is a single center study with patients recruited at the regional Hospital of Oberwart that serves a region of roughly 60.000 people. Patients were recruited from 2011 to 2013 and were included in the study if they participated in the 12 months follow up. All patients fulfilled the suggested guidelines for bariatric surgery as published previously [3]. Women who turned pregnant during follow-up, patients below 18 years of age and patients with known malignant disease or diabetes mellitus type 1 were excluded from the study. Venous blood was drawn the day before surgery and after 12 months. All blood samples were centrifuged (2800 rpm, 20 min) and frozen in aliquots. All samples were stored at − 80 °C before analysis.

### Prothrombin time (PT) and activated partial thromboplastin time (aPTT) determination

Both PT and aPTT where measured on a Siemens BCS XP system using commercially available assays pathromtin SL for aPTT and thromborel S for PT (both Siemens, Germany) as suggested by the manufacturer.

### Protein determination

Total PC was measured in plasma from citrate anticoagulated blood using a commercially available ELISA kit as suggested by the manufacturer’s instruction (Technoclone, Austria). A pooled standard from 10 randomly selected samples (five pre-surgery, five from the 12 month time point) was used to obtain a reference value. APC was assessed in citrated plasma samples using a chromogenic assay for measuring PC (Actichrome ©, Sekisui Diagnostics, Germany). The same pooled plasma standard was used as for the total PC ELISA. TM plasma levels were determined in citrated plasma samples using a commercially available ELISA (BioVendor, Czech Republic) following the suggested protocol. Soluble E-selectin (E-Sel) was analyzed using an ELISA (Thermo Fisher, USA) following the manufacturer’s instructions. Concentrations of high sensitive CRP (hs-CRP) were measured using particle enhanced immunoturbidimetric assay on cobas® 8000 modular analyzer (Cardiac C-Reactive Protein (Latex) High Sensitive, Roche Diagnostics, Switzerland). PAI-1 levels were determined by an ELISA as suggested by the manufacturer (Technoclone).

### Statistics

For statistical analysis SPSS21 (IBM, USA) was used. After an initial Kolmogorov–Smirnov test did not demonstrate normal distribution for all parameters statistical significance of differences between time points were calculated using Wilcoxon Ranks Test with significance assumed at p ≤ 0.05.

## Results

We studied changes in coagulatory parameters in morbidly obese patients undergoing Roux-en-Y gastric bypass surgery. Our study cohort consisted of 60 individuals, 19 male (32%) and 41 female patients with a mean age of 40.8 ± 12.5. One year after gastric bypass surgery, patients showed significant reduction of weight, BMI, total cholesterol, total LDL, high sensitive IL-6, high sensitive CRP, and of the prothrombotic adipokine plasminogen activator inhibitor-1 (PAI-1) and a significant increase in total HDL (Table 1). In addition, patients had reduced medication intake (Table 2).

**Table 1:** Baseline characteristics of patients before and after bariatric surgery

	Before surgery	1 year after surgery	p-value
Weight (kg)	126.5 ± 16.7	79.8 ± 14	< 0.001
BMI	43.7 ± 3.7	27.5 ± 3.8	< 0.001
Total cholesterol (mg/dl)	180.4 ± 36.8	150.3 ± 29.6	< 0.001
Total LDL (mg/dl)	101.6 ± 23.2	76.7 ± 20.9	< 0.001
Total HDL (mg/dl)	45.7 ± 11.4	54.5 ± 11.9	< 0.001
high sensitive IL-6 (pg/ml)	3.1 ± 2.5	1.7 ± 1.3	< 0.001
high sensitive CRP (mg/dl)	0.9 ± 1.6	0.2 ± 0.77	< 0.001
PAI-1 (pg/ml)	99.9 ± 16.3	83.3 ± 26.1	< 0.001

Patient characteristics are given for patients before and 1 year after bariatric surgery. Values are given as mean ± standard deviation, p < 0.05 was considered significant

**Table 2:** Medication use before and after bariatric surgery

Medication	Before surgery	1 year after surgery
Statin	14 (23%)	5 (8%)
Antidiabetics	14 (23%)	3 (5%)
Insulin	4 (7%)	3 (5%)
ACE inhibitors	26 (43%)	14 (23%)
Beta blocker	15 (25%)	5 (8%)

Medication use is given as patients receiving given medication with percentage of total patient population given in parenthesis

**Fig. 1:**
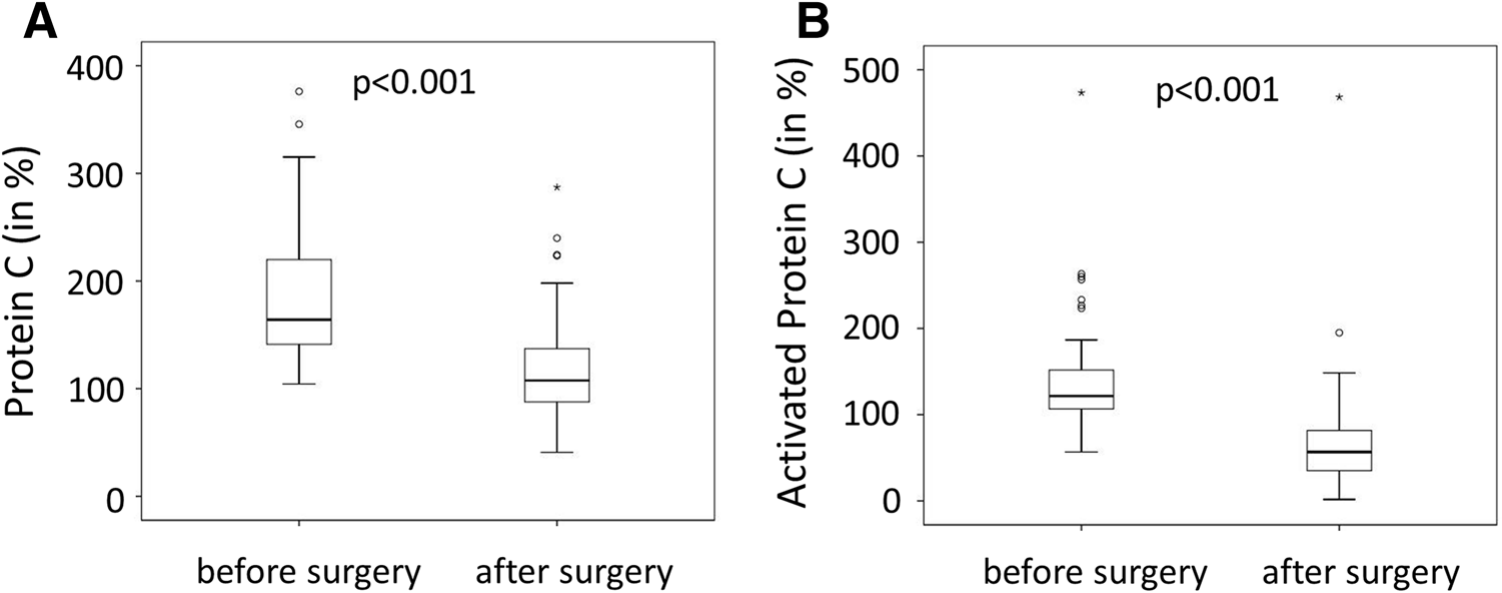
Protein C and activated protein C plasma levels before and 1 year after bariatric surgery. Protein C (**a**) and activated protein C (**b**) were measured using specific ELISAs as indicated under Methods in 60 patients before and 1 year after bariatric surgery. Values are given in percent compared to a pooled plasma standard. p < 0.05 was considered significant

To analyze the regulation of the natural anticoagulant PC and its activation, we evaluated plasma levels of total PC and APC. Values of PC and APC are given in relation to a pooled reference standard dilution series as described in the Methods section. Total protein C was reduced by 50% from 187.4 ± 64.5% before surgery to 118.1 ± 48% 1 year after surgery (Fig. 1a). Similarly, APC values were cut in half from 138.7 ± 64.4 to 69.1 ± 65.7% (Fig. 1b). Of note, changes in PC, APC and BMI were not significantly dependent on gender or diabetes as determined in a multivariate analysis.

**Fig. 2:**
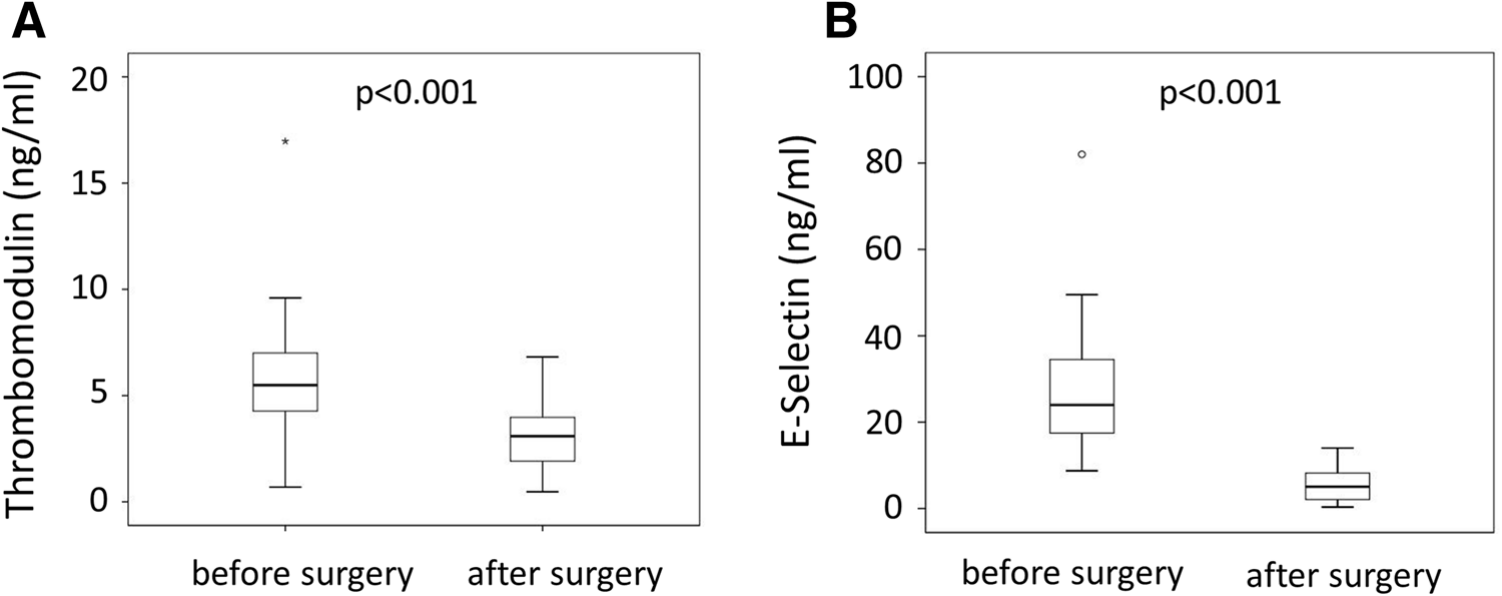
Soluble thrombomodulin plasma levels before and 1 year after bariatric surgery. Soluble thrombomodulin (**a**) and soluble E-selectin (**b**) were determined in plasma from 60 patients before and 1 year after bariatric surgery using specific ELISAs as indicated under Methods. Values are given in ng/ml. p < 0.05 was considered significant

The activation of protein C is driven by TM present on vascular endothelial cells. In contrast soluble TM is associated with an increased risk of thrombosis [18]. We observed increased levels of thrombomodulin prior to bariatric surgery with levels dropping from an initial 5.7 ± 2.6 to 3.2 ± 1.4 ng/ml after surgery (Fig. 2). Besides activating protein C, TM is also associated with endothelial cell activation. In addition to TM we measured a second marker of endothelial activation, soluble E-Sel. Plasma levels of soluble E-Sel dropped 1 year after bariatric surgery (26.6 ± 12.7 ng/ml before surgery versus 5.5 ± 4.1 ng/ml after surgery, p < 0.001). Similar to previous studies, soluble ICAM did not show a change after surgery [19] (data not shown).

**Fig. 3:**
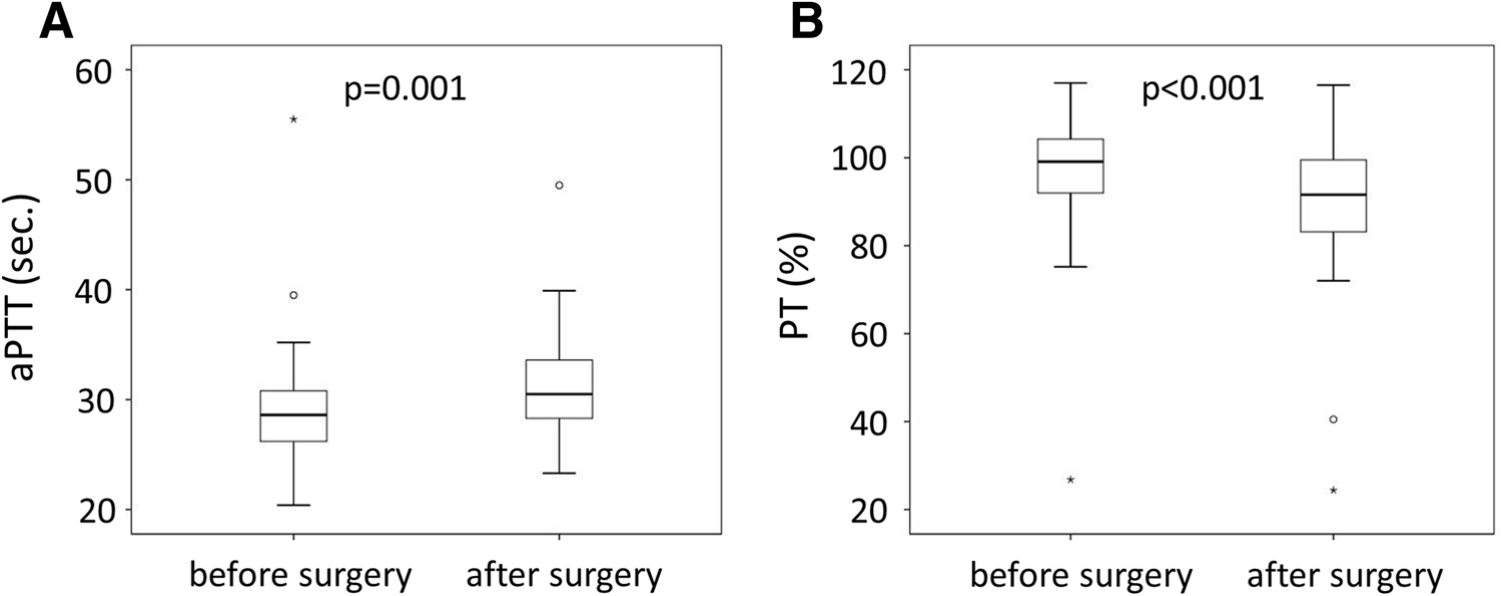
Activated partial thromboplastin time and prothrombin time in patients before and 1 year after bariatric surgery. Activated partial thromboplastin time (aPTT, **a**) was evaluated in 54 patients before and 1 year after bariatric surgery. Prothrombin time (PT, **b**) was determined in 52 patients before and 1 year after bariatric surgery. p < 0.05 was considered significant

Overall changes in the coagulatory capacity before and after surgery in patients were determined by aPTT. Values were available for 54 patients for both time points. We found a small but significant increase of the aPTT time from an average of 29.1 ± 4.8 s. before surgery to 31 ± 4.4 s. after surgery (Fig. 3a). In contrast PT, which was available for 52 patients, was slightly but significantly reduced in patients after bariatric surgery from 97.5 ± 13.5 to 89.6 ± 15.5% (Fig. 3b).

## Discussion

Overall, several parameters determined in our patient cohort were comparable to already established results from bariatric surgery patients including a massive drop of BMI, change in lipid profile including a drop in LDL and an increase in HDL and a reduction in inflammatory markers IL-6 and CRP and the prothrombotic protein PAI-1 [20, 21]. Similar drops in weight and CRP levels were also described in a Mexican cohort of bariatric surgery patients [22]. The overall amelioration of obesity was further met by a reduction in medication needed. We have found a significant reduction of PC, APC, soluble TM and soluble E-Sel in patients 1 year after bariatric surgery. Overall, however, changes in PC and APC did not impact on coagulation as aPTT was not shortened but slightly increased. In addition, PT showed a reduction 12 months after surgery.

This study was planned as an observational study. Due to the study design several limitations should be addressed. We did not enroll a control group partially because the gastric bypass surgical procedure leads to a massive drop in weight usually not seen without surgery. The surgical procedure itself reduces the size of the stomach to a small pouch and changes to some extent the uptake of nutrients. Therefore, observed changes might be due to weight loss or due to a combination of weight loss and surgical intervention. Furthermore, no patient reached a significant clinical endpoint associated with thrombotic complications (e.g. venous thromboembolism).

Previous reports suggested an increased PC and APC in obese patients which was reduced under supervised weight loss after 2 months [13]. The weight loss was however only moderate (BMI 46.3–42.3) as no surgical procedure was used. The decrease of PC observed in the previous work was statistically only significant in the patient group with the highest weight loss. Our cohort is characterized by a massive weight loss and a longer observation period. We can confirm the previously obtained data and extend the finding that massive weight loss reduces PC 1 year after bariatric surgery. Similar observations can be made for APC.

TM bound to endothelial cells is the major factor of activation of PC [23]. Soluble TM can be used as a readout for endothelial dysfunction as TM is shed from the endothelial cell surface under stress [18]. In addition soluble TM is associated with increased thrombotic risk [24]. Our data indicate reduced shedding of TM 1 year after bariatric surgery. Previous data suggested that TM cleavage is mainly caused by oxidative stress and not inflammation [25]. In line with this finding, a previous report demonstrated reduced oxidative stress after bariatric surgery [3]. To further analyze the possible effect of weight loss after bariatric surgery on endothelial cells we studied soluble E-Sel as a possible marker for endothelial cell activation. Previous reports suggested that E-Sel was reduced in obese patients after caloric restrictions [26] and is affected by bariatric surgery already after 4 months [19]. Our study supports previous findings and we describe a massive drop 1 year after surgery. We suggest that the drop in TM and soluble E-Sel levels reflects the reduced activation of the endothelium thereby also reducing overall thrombosis risk in patients after bariatric surgery.

Even though we measured reduced circulating levels of PC and APC, aPTT was slightly but significantly longer 1 year after bariatric surgery. aPTT depends on several factors including PC, factor VIII, and factor XII [27]. All of these factors have been already linked to obesity and factor XII was already shown to be reduced 2 months after bariatric surgery [28, 29]. Whether the upregulation of PC and APC during obesity is an anticoagulatory protective mechanism cannot be answered with our study design as proper control groups without surgery where not available within our hospital.

PC is vitamin K dependent [30]. Previously, it was suggested that a possible reason of PC reduction after bariatric surgery is due to a vitamin K dysregulation caused by malabsorption due to the surgical procedure [29]. We therefore measured PT, as this reflects the activities of the vitamin K dependent factors VII, X and II [31]. Furthermore, a strong correlation of factor VII activity with activity of PC, was shown [27]. Our data demonstrate a reduction of PT, which might indirectly point towards a possible vitamin K deficiency in patients after bariatric surgery. However, as PT values were not outside of the normal range even 1 year after bariatric surgery a pathological change in vitamin K dependent coagulation parameters can be excluded. It should be mentioned, that the lack of measurements of vitamin K levels before and after bariatric surgery constitute a limitation of our study.

In conclusion, we demonstrate a reduction of PC and APC 1 year after bariatric surgery, which is also accompanied by a reduction in soluble TM and soluble E-Sel. The latter might reflect an improvement of endothelial function due to the reduction of the overall inflammatory state as indicated by the decrease of the inflammatory markers IL6 and CRP. Interestingly the reduction in PC and APC is not paralleled by a reduction but in contrast by a prolongation of aPTT and a reduction in PT.
